# 17(*S*),18(*R*)‐epoxyeicosatetraenoic acid generated by cytochrome P450 BM‐3 from *Bacillus megaterium* inhibits the development of contact hypersensitivity via G‐protein‐coupled receptor 40‐mediated neutrophil suppression

**DOI:** 10.1096/fba.2019-00061

**Published:** 2019-12-24

**Authors:** Azusa Saika, Takahiro Nagatake, Shigenobu Kishino, Si‐Bum Park, Tetsuya Honda, Naomi Matsumoto, Michiko Shimojou, Sakiko Morimoto, Prabha Tiwari, Eri Node, So‐ichiro Hirata, Koji Hosomi, Kenji Kabashima, Jun Ogawa, Jun Kunisawa

**Affiliations:** ^1^ Laboratory of Vaccine Materials Center for Vaccine and Adjuvant Research Laboratory of Gut Environmental System National Institutes of Biomedical Innovation Health and Nutrition (NIBIOHN) Osaka Japan; ^2^ Graduate School of Pharmaceutical Sciences Osaka University Osaka Japan; ^3^ Division of Applied Life Sciences Graduate School of Agriculture Kyoto University Kyoto Japan; ^4^ Department of Dermatology Graduate School of Medicine Kyoto University Kyoto Japan; ^5^ Graduate School of Medicine Kobe University Hyogo Japan; ^6^ International Research and Development Center for Mucosal Vaccines The Institute of Medical Science The University of Tokyo Tokyo Japan; ^7^ Graduate School of Medicine Graduate School of Dentistry Osaka University Osaka Japan

**Keywords:** anti‐inflammation, dermatitis, epoxy‐fatty acid, lipid mediators, structure‐activity relationship

## Abstract

Dietary intake of ω3 polyunsaturated fatty acids such as eicosapentaenoic acid and docosahexaenoic acid is beneficial for health control. We recently identified 17,18‐epoxyeicosatetraenoic acid (17,18‐EpETE) as a lipid metabolite endogenously generated from eicosapentaenoic acid that exhibits potent anti‐allergic and anti‐inflammatory properties. However, chemically synthesized 17,18‐EpETE is enantiomeric due to its epoxy group—17(*S*),18(*R*)‐EpETE and 17(*R*),18(*S*)‐EpETE. In this study, we demonstrated stereoselective differences of 17(*S*),18(*R*)‐EpETE and 17(*R*),18(*S*)‐EpETE in amelioration of skin contact hypersensitivity and found that anti‐inflammatory activity was detected in 17(*S*),18(*R*)‐EpETE, but not in 17(*R*),18(*S*)‐EpETE. In addition, we found that cytochrome P450 BM‐3 derived from *Bacillus megaterium* stereoselectively converts EPA into 17(*S*),18(*R*)‐EpETE, which effectively inhibited the development of skin contact hypersensitivity by inhibiting neutrophil migration in a G protein‐coupled receptor 40‐dependent manner. These results suggest the new availability of a bacterial enzyme to produce a beneficial lipid mediator, 17(*S*),18(*R*)‐EpETE, in a stereoselective manner. Our findings highlight that bacterial enzymatic conversion of fatty acid is a promising strategy for mass production of bioactive lipid metabolites.

**Abbreviations**
17,18‐EpETE: 17,18‐epoxyeicosatetraenoic acid
18‐HEPE: 18‐hydroxyeicosapentaenoic acid
AhR: aromatic hydrocarbon receptor
CHS: contact hypersensitivity
CYP: cytochrome P450
DC: dendritic cell
DHA: docosahexaenoic acid
DNFB: 1‐fluoro‐2,4‐dinitrofluorobenzene
EPA: eicosapentaenoic acid
fMLP: N‐formyl‐methionyl‐phenylalanine
GPR: G protein‐coupled receptor
HE: hematoxylin and eosin
HPLC: high performance liquid chromatography
IFN‐γ: interferon‐γ
IL: interleukin
LTB_4_: leukotriene B_4_
PE: phycoerythrin
PUFA: polyunsaturated fatty acid
RvE1: resolvin E1
SPM: specialized pro‐resolving mediator
WT: wild‐type

## 1 INTRODUCTION

ω3 polyunsaturated fatty acids (PUFAs), such as eicosapentaenoic acid (EPA) and docosahexaenoic acid (DHA), are well known to exert beneficial effects on various inflammatory diseases, including cardiovascular diseases, cancer, rheumatoid arthritis, ulcerative colitis, asthma, and atopic dermatitis.^1^, ^2^ However, the molecular mechanisms through which ω3 PUFAs exert such beneficial effects are not fully understood. Recent evidence has revealed that ω3 PUFAs are converted enzymatically and non‐enzymatically into specialized pro‐resolving mediators (SPMs), which show anti‐allergic and anti‐inflammatory activities. For example, resolvins, protectins, maresins, and 18‐hydroxyeicosapentaenoic acid (18‐HEPE) are representative SPMs that regulate inflammatory animal models such as colitis, peritonitis, asthma, and contact hypersensitivity (CHS).^3^, ^4^, ^5^, ^6^, ^7^, ^8^ We recently reported that 17,18‐epoxyeicosatetraenoic acid (17,18‐EpETE) is a new class of anti‐allergy and anti‐inflammatory lipid mediator that inhibits the development of food allergy and CHS.^9^, ^10^

The stereochemistry of oxidized fatty acids is a critical determinant of their biological activity. Indeed, 17,18‐EpETE is enantiomeric due to its epoxy groups—17(*S*),18(*R*)‐EpETE and 17(*R*),18(*S*)‐EpETE. One enantiomer may be responsible for the therapeutic effects, whereas the other enantiomer can be inactive or exert undesired effects. For example, 2(*S*)‐hydroxy oleic acid induces greater reduction in the tumor volume of lung cancer than does 2(*R*)‐hydroxy oleic acid.^11^ Furthermore, 17(*R*),18(*S*)‐EpETE—but not 17(*S*),18(*R*)‐EpETE—has vasodilatory effects on arteries, again suggesting that chirality is important for determination of biological activity.^12^, ^13^ However, which enantiomer of 17,18‐EpETE is responsible for anti‐inflammatory activity in the regulation of CHS remains unclear.

Although 17,18‐EpETE is endogenously generated from EPA by cytochrome P450 (CYP), microorganisms also harbor various types of CYPs which catalyze a wide range of reactions including epoxidation and hydroxylation.^9^, ^10^, ^14^ Previously, 18(*R*)‐HEPE which plays important roles as a SPM and a precursor of resolvin E1 (RvE1) was shown to be generated by hydroxylation of EPA using *Bacillus megaterium* homogenates.^15^ This finding suggested that bacterial enzymes were useful to produce various bioactive lipid mediators. In addition, it is reported that BM‐3, a CYP derived from *B. megaterium*, catalyzed the epoxidation of unsaturated fatty acids and converted EPA into 17(*S*),18(*R*)‐EpETE in a stereoselective manner,^16^, ^17^, ^18^ suggesting its potential for industrial applications in synthesizing epoxy compounds.^19^

Contact hypersensitivity is a commonly used mouse model of human allergic contact dermatitis, which includes sensitization and elicitation phases. In the sensitization phase, dendritic cells (DCs) migrate to the draining lymph nodes and activate T cells for the induction of memory‐type T cells. In the elicitation phase, on exposure to the same contact allergen as experienced during the sensitization phase, neutrophils and memory‐type T cells infiltrate the inflamed skin, where they produce pro‐inflammatory cytokines that lead to the development of skin swelling, rashes, and edema.^20^ Among the various steps in the development of CHS, 17,18‐EpETE suppresses neutrophil infiltration into inflamed skin by inhibiting neutrophil pseudopod formation in a G protein‐coupled receptor (GPR) 40‐dependent manner.^10^

In this study, we found that 17(*S*),18(*R*)‐EpETE produced by BM‐3 (BM‐3 17(*S*),18(*R*)‐EpETE) ameliorated CHS by inhibiting neutrophil migration into inflamed skin in a GPR40‐dependent manner. These findings reveal the activity of the 17(*S*),18(*R*)‐EpETE enantiomer and the availability of a bacterial enzyme that produces this bioactive lipid metabolite in a stereoselective manner.

## 2 MATERIALS AND METHODS

### 2.1 Animals

Wild‐type (WT) C57BL/6J female mice (age, 6‐8 weeks) were purchased from SLC (Shizuoka, Japan) and kept in a specific pathogen‐free animal facility at the National Institutes of Biomedical Innovation, Health and Nutrition (NIBIOHN) for at least 1 week before use in experiments. GPR40‐deficient mice have been described previously^21^ and were bred and maintained in the animal facility at NIBIOHN. For euthanasia, mice were deeply anesthetized using isoflurane (Forane, AbbVie) and then killed through cervical dislocation.

### 2.2 Induction of CHS

Contact hypersensitivity was induced as described previously.^10^ Briefly, the abdominal skin of mice was shaved, after which 25 μL of 0.5% (vol/vol) 1‐fluoro‐2,4‐dinitrofluorobenzene (DNFB, Nacalai Tesque) dissolved in a mixture of acetone (Nacalai Tesque) and olive oil (Nacalai Tesque) at a ratio of 4:1 was applied. After 5 days, both sides of the right and left ears were challenged with 0.2% (vol/vol) DNFB (10 μL at each site). After another 2 days, ear thickness was measured using a micrometer (model MDC‐25MJ 293‐230, Mitsutoyo). In order to evaluate the effects of 17,18‐EpETE, mice received racemic compound of 17(*S*),18(*R*)‐EpETE and 17(*R*),18(*S*)‐EpETE ((±)17,18‐EpETE), a commercially available Cayman (±)17,18‐EpETE (Cayman Chemical). In some experiments, mice were treated with stereoselective 17(*S*),18(*R*)‐EpETE (>99% enantiomeric excess) or 17(*R*),18(*S*)‐EpETE (>99% enantiomeric excess), which were purified from synthesized (±)17,18‐EpETE, or BM‐3 17(*S*),18(*R*)‐EpETE. These lipids were injected intraperitoneally into mice by 100 ng/animal at 30 minutes before DNFB treatment. In some experiments, BM‐3 17(*S*),18(*R*)‐EpETE were injected intraperitoneally into mice by 1 µg, 100 ng, or 10 ng/animal in order to evaluate dose response. We used 0.5% (vol/vol) ethanol dissolved in PBS (Nacalai Tesque) as a vehicle control.

### 2.3 Preparation of 17(*S*),18(*R*)‐EpETE and 17(*R*),18(*S*)‐EpETE

The (±)17,18‐EpETE was prepared from EPA as described previously with some modifications.^22^ In brief, 3.5 g of EPA was reacted with 1.96 g of carbonyl diimidazole (Tokyo Chemical Industry Co. Ltd) in 15 mL of dry dichloromethane (Wako) with stirring for 3 hours at room temperature under nitrogen. The resultant mixture was added slowly over 5 minutes into ethereal hydroperoxide (Wako) on ice for EPA‐epoxidation. The epoxide product was extracted by diethyl ether (Kishida Chemical Co. Ltd) and washed with water. After removing solvent in vacuo, the synthesized (±)17,18‐EpETE was purified by reverse‐phase high performance liquid chromatography (HPLC) equipped with a Cosmosil Cholester column (250 × 10 mm, 5 μm, Nacalai Tesque). The isocratic mobile phase was a mixture of acetonitrile (Wako) and water containing 0.1% (vol/vol) formic acid (Wako) in 60:40 (vol/vol), which was pumped at a flow rate of 5 mL/min. The column was maintained at 30°C, and the eluent was monitored at a wavelength of 205 nm. The chemical structure of synthesized 17,18‐EpETE was confirmed by NMR analysis.

For purification of stereoselective 17,18‐EpETE, HPLC was performed using a CHIRALCEL OJ‐RH packed column (150 × 4.6 mm, 5 μm, Daicel). The isocratic mobile phase was a mixture of methanol (Wako) and water containing 0.1% (vol/vol) formic acid (75:25, vol/vol), which was pumped at a flow rate of 1.2 mL/min. The column was maintained at 30°C, and the eluent was monitored at a wavelength of 205 mm. As a control, commercially available Cayman (±)17,18‐EpETE was used for HPLC analysis.

### 2.4 Construction of BM3‐expressing plasmid and transformation of *Escherichia coli*

BM3‐encoding pFusionF87V plasmid was ligated into pET‐24d (+) (Merck) at KNC Laboratories (Hyogo, Japan).^23^ The gene was suspended with ECOS Competent *E. coli* DH5α (Nippon Gene), and transformed by heat shock at 42°C for 30 seconds. After the transformation, *E. coli* DH5α was inoculated to modified LB agar medium (animal‐derived material‐free) containing 25 μg/mL kanamycin sulfate (Wako) and cultured at 37°C for 18 hours. Modified LB agar medium was comprised of 1% (wt/vol) Difco select soytone (Becton, Dickinson and Company), 0.5% (wt/vol) Bacto yeast extract (Becton, Dickinson and Company), 1% (wt/vol) sodium chloride (Wako) and 1.5% (wt/vol) agar powder (Wako). The resultant colonies were picked and inoculated to modified LB liquid culture medium (animal‐derived material‐free) containing 25 μg/mL kanamycin sulfate and cultured at 37°C for 18 hours. Modified LB liquid culture medium was comprised of 1% (wt/vol) Difco select soytone, 0.5% (wt/vol) Bacto yeast extract and 1% (wt/vol) sodium chloride. The plasmid DNA was extracted using QIAprep Spin Miniprep Kit (Qiagen) according to the manufacturer's instructions. In this way, pFusionF87V‐Km plasmid was constructed.

We next used pFusionF87V‐Km plasmid as a template for construction of pFusionBM3‐WT plasmid to give rise to large amount of 17,18‐EpETE. The primers for inverse PCR were as follows: primer 1, 5'‐TTTACAAGCTGGACGCATGA‐3' and primer 2, 5'‐TAACCCGTCTCCTGCAAAATCAC‐3'. The fragments were self‐ligated using Ligation‐Convenience kit after phosphorylation of 5' ends by T4 polynucleotide kinase (Takara Bio). The resultant solution was suspended with ECOS Competent *E. coli* DH5α and transformed by heat shock at 42°C for 30 seconds. The recombinant *E. coli* was inoculated to modified LB agar medium (animal‐derived material‐free) containing 25 μg/mL kanamycin sulfate and cultured at 37°C for 18 hours. The resultant colonies were inoculated to modified LB liquid culture medium (animal‐derived material‐free) containing 25 µg/mL kanamycin sulfate and cultured at 37°C for 18 hours. The plasmid DNA was extracted using QIAprep Spin Miniprep Kit. In this way, pFusionBM3‐WT plasmid which has no mutation was constructed.

In order to carry pFusionBM3‐WT into *E. coli* BL21 (DE3) which is expression host strain, the pFusionBM3‐WT was suspended with ECOS Competent *E. coli* BL21 (DE3) (Nippon Gene) and transformed by heat shock at 42°C for 30 seconds. After the transformation, *E. coli* BL21 (DE3) was inoculated to modified LB agar medium (animal‐derived material‐free) containing 25 μg/mL kanamycin sulfate and cultured at 37°C for 18 hours. The resultant colonies were inoculated to modified LB liquid culture medium (animal‐derived material‐free) containing 25 µg/mL kanamycin sulfate and cultured at 28°C for 18 hours. The glycerol stock of pFusionBM3‐WT/BL21 (DE3) was made by mixing cultured solution and 50% (vol/vol) glycerol (Wako) in 2:1 (vol/vol) and stored at −20°C.

### 2.5 Cultivation of pFusionBM3‐WT/BL21 (DE3)

Cultivation was performed at KNC Laboratories. For preculture, 100 µL of pFusionBM3‐WT/BL21 (DE3) glycerol stock was inoculated in 500 mL of modified LB liquid culture medium (animal‐derived material‐free) containing 25 µg/mL kanamycin sulfate and cultured at 25°C for 22 hours with shaking at 120 rpm.

1 L of precultured liquid was added to 150 L of modified 2 × YT medium (animal‐derived material‐free) containing 25 µg/mL kanamycin sulfate, 80 µg/mL 5‐aminolevulinic acid (Wako), 100 µM ammonium iron (II) sulfate hexahydrate (Wako), 250 µmol/L isopropyl β‐_D_‐thiogalactopyranoside (IPTG; Wako) and cultured at 20°C for 47 hours with ventilation rate of 75 L/min. 2 × YT medium was comprised of 1.6% (wt/vol) Difco select soytone, 1% (wt/vol) Bacto yeast extract and 0.5% (wt/vol) sodium chloride. pH in culture was maintained at pH 7.0 ± 0.1 using 25% (vol/vol) ammonia solution (Wako) and 2 mol/L phosphoric acid (Wako), and dissolved oxygen was maintained at DO 1.5 ± 0.5 ppm by stirring.

### 2.6 Bioconversion of EPA into 17,18‐EpETE by pFusionBM3‐WT/BL21 (DE3)

Bioconversion of EPA into 17,18‐EpETE was performed at KNC Laboratories. 1.5 L of 1 mol/L EPA (Carbosynth, Berkshire, UK) was added to cultured medium and incubated at 20°C for 71.5 hours with ventilation rate of 20 L/min in order to convert EPA into 17,18‐EpETE. pH was maintained at pH 7.0 ± 0.1 using 25% (vol/vol) ammonia solution and 2 mol/L phosphoric acid, and dissolved oxygen was maintained at DO 1.5 ± 0.5 ppm by stirring.

In order to stop reaction and kill bacteria, 35 L of ethanol (Wako) was added to reaction mixture and cultured at 20°C for 46 hours with ventilation rate of 20 L/min. pH was maintained at pH 7.0 ± 0.1 using 25% (vol/vol) ammonia solution and 2 mol/L phosphoric acid, and dissolved oxygen was maintained at DO 1.5 ± 0.5 ppm by stirring. In order to confirm whether the bacteria are dead, the reaction liquid was inoculated to modified LB agar medium (animal‐derived material‐free) and cultured at 37°C for 18 hours, then we confirmed that the colonies were not formed.

### 2.7 Purification of BM‐3 17(*S*),18(*R*)‐EpETE

Purification of BM‐3 17(*S*),18(*R*)‐EpETE was performed at KNC Laboratories. Fifteen kilograms of Diaion HP20 (Mitsubishi Chemical) were added to 150 L of EPA reaction solution (20% (vol/vol) ethanol aqueous solution) and stirred for 1 hour to adsorb 17,18‐EpETE, which was confirmed by the analysis of the supernatant with HPLC Prominence System (Shimadzu). The condition of HPLC analysis is as follows: flow rate; 1.0 mL/min, column temperature; 40°C, UV wavelength; 205 nm, injection volume; 10 µL, column; C_18_ column (Kinetex 5u C18 100A column, 100 × 4.6 mm ID, Phenomenex), mobile phase; (A) 0.05% (vol/vol) formic acid aqueous solution and (B) acetonitrile. The eluent gradients were 45%‐55% (vol/vol) B for 0‐12 minutes, 55%‐75% (vol/vol) B for 12‐19 minutes, 75%‐100% (vol/vol) B for 19‐20 minutes, 100% (vol/vol) B for 20‐28 minutes, 100%‐45% (vol/vol) B for 28‐29 minutes, and 45% (vol/vol) B for 29‐40 minutes. After the absorption of 17,18‐EpETE, HP20 was removed from the EPA reaction solution, and washed four times with purified water. Then, HP20 was soaked in 20% (vol/vol) ethanol aqueous solution and stored at 4°C.

In order to isolate 17,18‐EpETE from HP20, 200 mL of ethanol were added to 174 g of HP20 and stirred for 10‐15 minutes, followed by filtration under reduced pressure and resuspended in 200 mL of ethanol. The same operations of filtration and suspension in ethanol were repeated five times.

In order to purify 17,18‐EpETE, resultant ethanol solution containing 17,18‐EpETE was diluted to five times with Milli‐Q water containing acetic acid (Wako) (final concentration; 20% (vol/vol) ethanol aqueous solution containing 0.05% (vol/vol) acetic acid). This solution was charged to Inertsil ODS‐3 column (250 × 50 mm ID, 5 µm, GL Sciences Inc) that had been equilibrated with 20% (vol/vol) ethanol aqueous solution containing 0.05% (vol/vol) acetic acid at 50 mL/min flow rate. After charged, the column was washed with 20% (vol/vol) ethanol aqueous solution containing 0.05% (vol/vol) acetic acid, and eluted with 70% (vol/vol) ethanol aqueous solution containing 0.05% (vol/vol) acetic acid at 45 mL/min flow rate. The elution sample containing 17,18‐EpETE was stored at −80°C as a low purity sample (purity; more than 70%).

In order to obtain high purity sample, the low purity sample was charged to Inertsil ODS‐3 column (250 × 20 mm ID, 5 µm, GL Sciences Inc) that had been equilibrated with 20% (vol/vol) ethanol aqueous solution containing 0.05% (vol/vol) acetic acid at 15 mL/min flow rate. After charged, the column was washed with 20% (vol/vol) ethanol aqueous solution containing 0.05% (vol/vol) acetic acid, and eluted with 55% (vol/vol) ethanol aqueous solution containing 0.05% (vol/vol) acetic acid at 10 mL/min flow rate. The elution sample containing 17,18‐EpETE was stored at −80°C as a high purity sample (purity; more than 90%).

In order to replace the solvent to ethanol, the elution sample containing 17,18‐EpETE was diluted with Milli‐Q water containing acetic acid (final concentration; 20% (vol/vol) ethanol aqueous solution containing 0.05% (vol/vol) acetic acid). The resultant solution was charged to Inertsil ODS‐3 column (250 × 20 mm ID, 5 µm) that had been equilibrated with 20% (vol/vol) ethanol aqueous solution containing 0.05% (vol/vol) acetic acid at 15 mL/min flow rate. After charged, the column was washed with 20% (vol/vol) ethanol aqueous solution containing 0.05% (vol/vol) acetic acid, followed by washed with 20% (vol/vol) ethanol aqueous solution, and eluted with 100% (vol/vol) ethanol at 15 mL/min flow rate. The elution sample containing 17,18‐EpETE was stored at −80°C as a very high purity sample (purity; more than 95%), and used as BM‐3 17(*S*),18(*R*)‐EpETE for biological assay.

### 2.8 Histologic analysis

Histologic analysis was performed as described previously.^10^ In brief, ear samples were washed with PBS, embedded in Tissue‐Tek OCT Compound (Sakura Finetek), and frozen in liquid nitrogen. Frozen tissue sections (6 µm) were prepared using a cryostat (model CM3050 S, Leica Biosystems) at –20°C.

For hematoxylin and eosin (HE) staining, frozen tissue sections were stained in hematoxylin solution (Wako) for 10 minutes and washed with water for 30 minutes. Then, the sections were stained in 1% eosin Y solution (Wako) for 1 minute and dehydrated through increasing concentrations of ethanol (70% to 100%; Nacalai Tesque). Finally, stained tissue sections were dehydrated in xylene (Nacalai Tesque) for 3 minutes and mounted in Permount (Falma).

For immunohistologic analysis, frozen tissue sections were washed with PBS for 10 minutes and then blocked in 2% (vol/vol) newborn calf serum (Equitech‐Bio) in PBS for 30 minutes at room temperature in an incubation chamber (Cosmo Bio). Tissue sections were then incubated in an incubation chamber overnight at 4°C with fluorescein isothiocyanate–anti‐Ly6G monoclonal antibody (dilution, 1:100; catalog no. 127606, BioLegend) in 2% (vol/vol) newborn calf serum in PBS. Then, samples were washed once for 5 minutes in 0.1% (vol/vol) Tween‐20 (Nacalai Tesque) in PBS and then in PBS only for 5 minutes. To visualize nuclei, tissue sections were stained with 4ʹ,6‐diamidino‐2‐phenylindole (1 μmol/L; AAT Bioquest) for 10 minutes at room temperature in the incubation chamber. Finally, tissue sections were washed twice with PBS for 5 minutes each, and mounted in Fluoromount (Diagnostic BioSystems) and examined under a fluorescence microscope (model BZ‐9000, Keyence).

### 2.9 Cell isolation and flow cytometric analysis

The isolation of cells from ear tissue and their flow cytometric analysis were performed as described previously.^10^ Ears were split and the cartilage removed using tweezers. The skin samples were macerated and then incubated in RPMI 1640 medium (Sigma Aldrich) containing 2% (vol/vol) newborn calf serum and 2 mg/mL collagenase (Wako) for 90 minutes at 37°C with stirring.

Cell suspensions were filtered using a cell strainer (pore size, 100 μm; BD Biosciences) and cells counted. Cells were stained using an anti‐CD16/32 monoclonal antibody (TruStain fcX; dilution, 1:100; catalog no. 101320, BioLegend) to avoid nonspecific staining, and dead cells were stained with 7‐aminoactinomycin D (dilution, 1:100; catalog no. 420404, BioLegend). The cells were further stained with the following antibodies: fluorescein isothiocyanate–anti‐Ly6G (dilution, 1:100), allophycocyanin–Cy7–anti‐CD11b (dilution, 1:100; catalog no. 101226, BioLegend), and BV421–anti‐CD45 (dilution, 1:100; catalog no. 103133, BioLegend). Samples were analyzed using MACSQuant (Miltenyi Biotec, Bergish Gladbach, Germany). Data were analyzed using FlowJo 9.9 software (TreeStar).

### 2.10 Purification of neutrophils from bone marrow and in vitro neutrophil assay

Neutrophils were purified from bone marrow as described previously.^10^ Briefly, bone marrow‐derived neutrophils were harvested using 62% Percoll. For the actin polymerization assay, purified neutrophils (4 × 10^5^ cells) were suspended in HBSS (Nacalai Tesque) containing 0.2% bovine serum albumin (Sigma Aldrich) and allowed to adhere to fibronectin‐coated coverslips (Neuvitro) for 15 minutes at 37°C in a 5% CO_2_ incubator. Neutrophils were treated with either 1000, 100, 10, or 1 nmol/L of commercially available Cayman (±)17,18‐EpETE, BM‐3 17(*S*),18(*R*)‐EpETE, 18‐HEPE (Cayman Chemical), RvE1 (Cayman Chemical), or 0.03% (vol/vol) ethanol (vehicle control) for 15 minutes and then stimulated with 1 μmol/L N‐formyl‐methionyl‐phenylalanine (fMLP; Sigma Aldrich) or 100 nmol/L leukotriene B_4_ (LTB_4_; Cayman Chemical) for 2 minutes at 37°C in a 5% CO_2_ incubator. Neutrophils were fixed in 4% paraformaldehyde (Nacalai Tesque), permeabilized using 0.5% (vol/vol) Triton X‐100 (Nacalai Tesque) in PBS, and stained with 100 nmol/L Acti‐stain 488–phalloidin (Cytoskeleton) for 30 minutes at room temperature. Finally, cell nuclei were stained by incubating neutrophils with 4ʹ,6‐diamidino‐2‐phenylindole for 30 seconds at room temperature. Images were obtained with Leica TCS SP8 confocal microscopy (Leica Microsystems).

### 2.11 Reverse transcription and quantitative PCR analysis

Reverse transcription and quantitative PCR analysis were performed as described previously.^10^ Ears were placed in XXTuff microvials (BioSpec Products) and crushed using stainless‐steel beads (4.8 φ and 3.2 φ, Tomy Digital Biology Co., Ltd) for 5 pulses (4800 rpm, 10 seconds each; Minibead‐beater, BioSpec Products) to isolate RNA from ear tissues. The samples were put on ice for 10 seconds between pulses. Samples were centrifuged, and the supernatant was processed to obtain total RNA. RNA was isolated using a Relia Prep RNA tissue miniprep system (Promega) following the manufacturer's instructions. Total RNA was incubated with DNase I (Invitrogen), and reverse transcribed into cDNA using Super Script VILO cDNA Synthesis kit (Invitrogen). Quantitative PCR analysis was performed with LightCycler 480 II (Roche) with FastStart Essential DNA Probes Master (Roche). Primer sequences were as follows: *Actinβ* sense, 5'‐aaggccaaccgtgaaaagat‐3'; *Actinβ* antisense, 5'‐gtggtacgaccagaggcatac‐3'; *interferon‐γ* (*Ifn‐γ*) sense, 5'‐atctggaggaactggcaaaa‐3'; *Ifn‐γ* antisense, 5'‐ttcaagacttcaaagagtctgaggta‐3'; *interleukin* (*Il*)*‐17* sense, 5'‐cagggagagcttcatctgtgt‐3'; *Il‐17* antisense, 5'‐gctgagctttgagggatgat‐3'; *Cxcl1* sense, 5'‐gactccagccacactccaac‐3'; *Cxcl1* antisense, 5'‐tgacagcgcagctcattg‐3'; *Cxcl2* sense, 5'‐aaaatcatccaaaagatactgaacaa‐3'; *Cxcl2* antisense, 5'‐ctttggttcttccgttgagg‐3'; *Cxcl9* sense, 5'‐cttttcctcttgggcatcat‐3'; *Cxcl9* antisense, 5'‐gcatcgtgcattccttatca‐3'; *Cxcl10* sense, 5'‐gctgccgtcattttctgc‐3'; *Cxcl10* antisense, 5'‐tctcactggcccgtcatc‐3'.

### 2.12 Statistics

Statistical significance was evaluated through one‐way ANOVA using Prism 3.03 software (GraphPad Software). A *P* < 0.05 was considered significant.

## 3 RESULTS

### 3.1 Synthesis of (±)17,18‐EpETE and analysis of 17(*S*),18(*R*)‐EpETE and 17(*R*),18(*S*)‐EpETE using HPLC system

**Figure 1:**
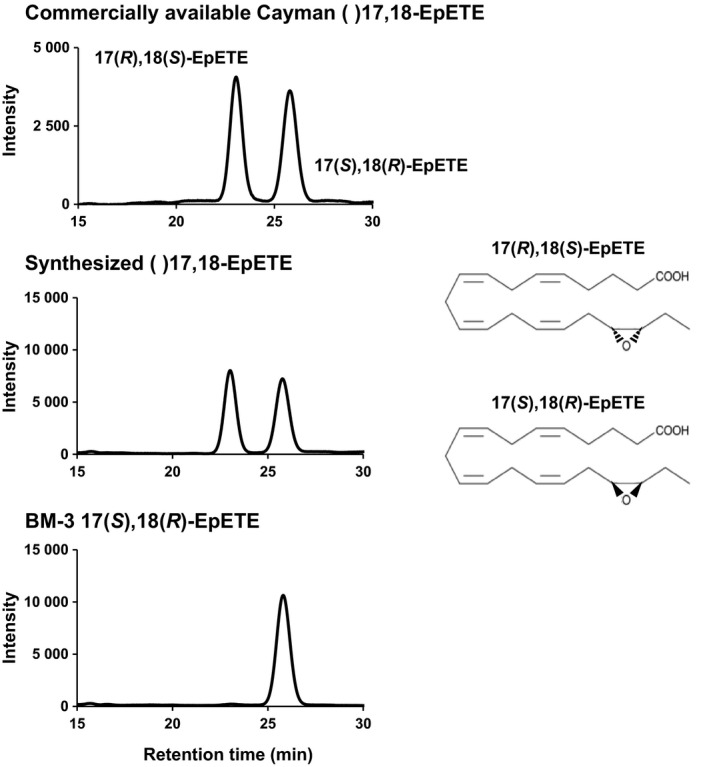
Synthesis of (±)17,18‐EpETE and analysis of 17,18‐EpETE enantiomers, 17(*S*),18(*R*)‐EpETE and 17(*R*),18(*S*)‐EpETE, using HPLC. Like the commercially available Cayman (±)17,18‐EpETE used as a standard, the synthesized (±)17,18‐EpETE detected two peaks using HPLC system with chiral column. Application of BM‐3 17(*S*),18(*R*)‐EpETE revealed that the second peak of (±)17,18‐EpETE corresponds to 17(*S*),18(*R*)‐EpETE, whereas the first peak is 17(*R*),18(*S*)‐EpETE

We previously reported that commercially available Cayman (±)17,18‐EpETE showed potent anti‐allergic and anti‐inflammatory properties.^9^, ^10^ To determine which enantiomer of 17,18‐EpETE shows anti‐inflammatory activity, we first sought to separate 17(*S*),18(*R*)‐EpETE from 17(*R*),18(*S*)‐EpETE. In this issue, we first chemically synthesized a large amount of (±)17,18‐EpETE from EPA and found that, like commercially available Cayman (±)17,18‐EpETE, synthesized (±)17,18‐EpETE showed 2 peaks using HPLC system with chiral column. The bacterial enzyme BM‐3 has been reported to stereoselectively convert EPA into 17(*S*),18(*R*)‐EpETE.^16^, ^24^ We found that BM‐3‐derived 17(*S*),18(*R*)‐EpETE was identical with the second peak of (±)17,18‐EpETE (Figure 1). Therefore, the first peak was regarded as 17(*R*),18(*S*)‐EpETE.

### 3.2 CHS is ameliorated by 17(*S*),18(*R*)‐EpETE but not 17(*R*),18(*S*)‐EpETE

**Figure 2:**
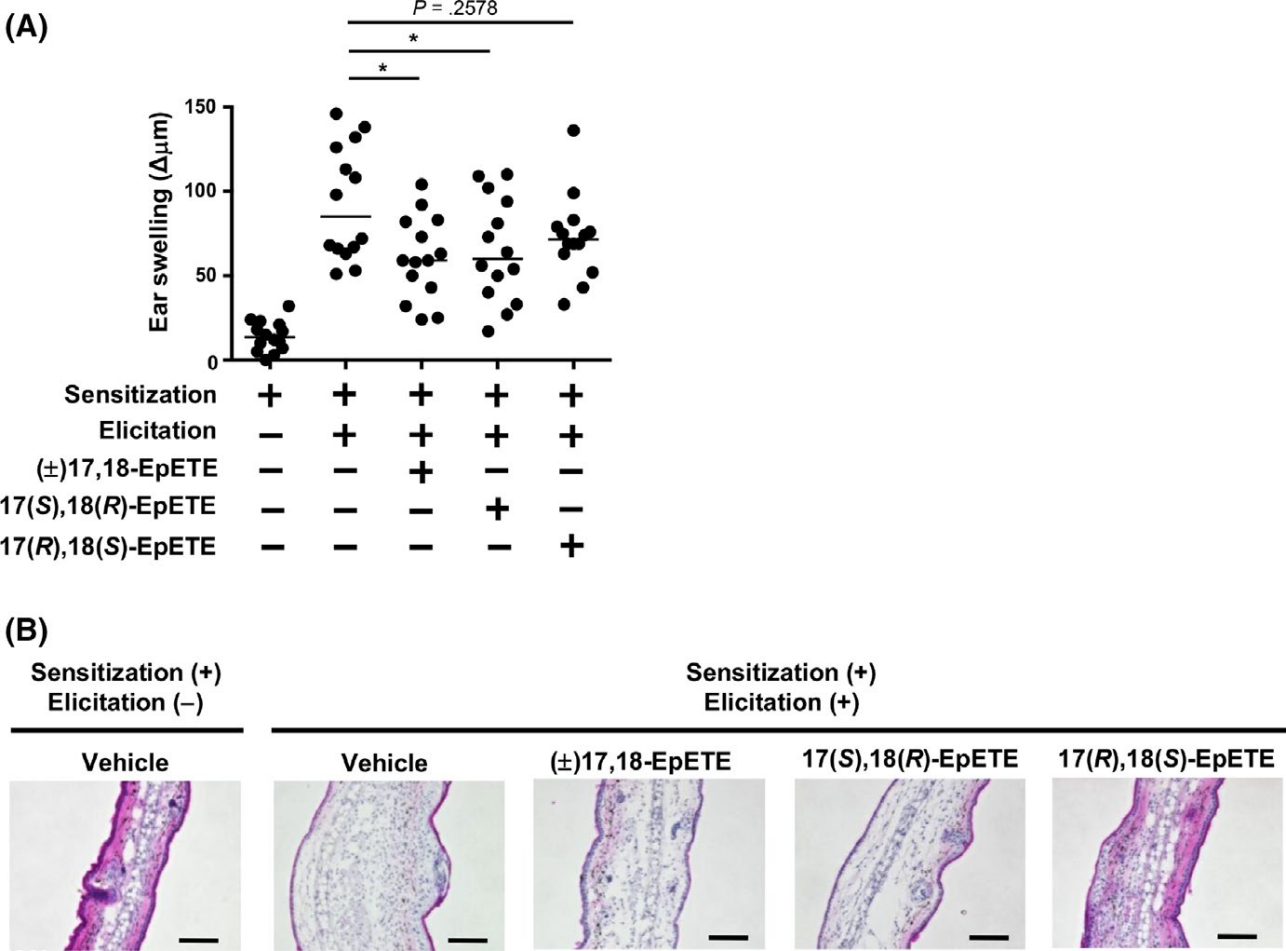
CHS is ameliorated by 17(*S*),18(*R*)‐EpETE. Mice were injected intraperitoneally with either commercially available Cayman (±)17,18‐EpETE, 17(*S*),18(*R*)‐EpETE, 17(*R*),18(*S*)‐EpETE, or vehicle only on days 0 and 5 at 30 min before DNFB application. A, Ear swelling was evaluated on day 7. Data are combined from three independent experiments, and each point represents data from an individual mouse. Horizontal bars among data points indicate median values. The statistical significance of differences between groups was evaluated using one‐way ANOVA: *P < .05. B, Ear tissue samples were prepared on day 7, stained with HE, and analyzed histologically. Data are representative of three independent experiments. Bars, 100 μm

We next examined the anti‐inflammatory effects of each 17,18‐EpETE enantiomer by using the mouse CHS model. We found that, like (±)17,18‐EpETE, 17(*S*),18(*R*)‐EpETE showed anti‐inflammatory activity in inhibiting ear swelling but 17(*R*),18(*S*)‐EpETE had little effect (Figure 2A). Histologic analysis yielded results similar to those from the ear swelling assay (Figure 2B).

### 3.3 BM‐3 17(*S*),18(*R*)‐EpETE ameliorates CHS by reducing neutrophil infiltration

**Figure 3:**
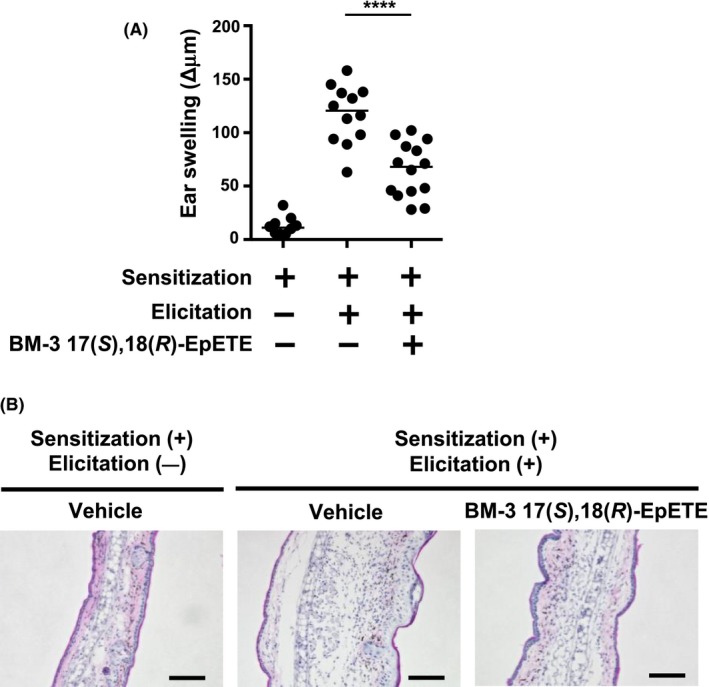
CHS is ameliorated by BM‐3 17(*S*),18(*R*)‐EpETE. Mice were injected intraperitoneally with either BM‐3 17(*S*),18(*R*)‐EpETE or vehicle only on days 0 and 5 at 30 min before DNFB application. A, Ear swelling was evaluated on day 7. Data are combined from three independent experiments, and each point represents data from an individual mouse. Horizontal bars among data points indicate median values. The statistical significance of differences between groups was evaluated by using one‐way ANOVA; ****P < .0001. B, Ear tissue samples were prepared on day 7, stained with HE, and analyzed histologically. Data are representative of three independent experiments. Bars, 100 μm

**Figure 4:**
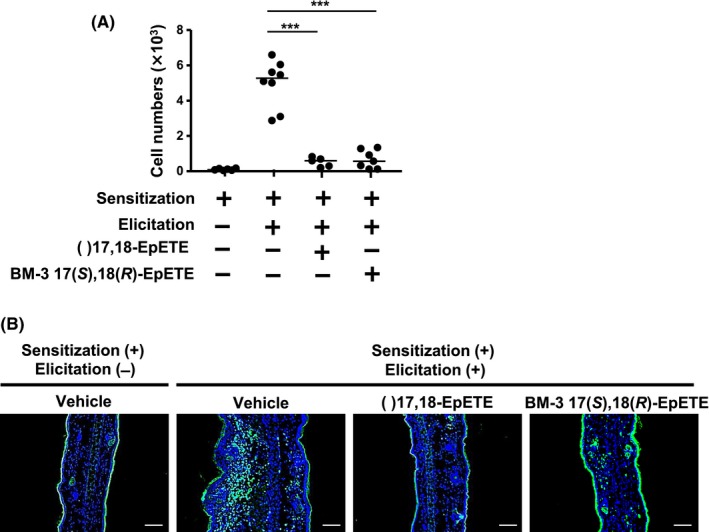
BM‐3 17(*S*),18(*R*)‐EpETE reduces the number of neutrophils. Mice were injected intraperitoneally with either commercially available Cayman (±)17,18‐EpETE, BM‐3 17(*S*),18(*R*)‐EpETE, or vehicle only on days 0 and 5 at 30 min before DNFB application. A, On day 7, flow cytometry was used to count the number of Ly6G^+^ CD11b^+^ neutrophils and calculate their proportion of the total cell count. Data are combined from two independent experiments, and each point represents data from an individual mouse. Horizontal bars among data points indicate median values. Statistical significance was evaluated using one‐way ANOVA; ***P < .001. B, Frozen ear sections obtained on day 7 were stained with fluorescein isothiocyanate‐labeled Ly6G monoclonal antibody and 4ʹ,6‐diamidino‐2‐phenylindole for immunohistologic analysis. Data are representative of two independent experiments. Bars, 100 μm

Given that 17(*S*),18(*R*)‐EpETE exerted anti‐inflammatory activity, we next examined whether 17(*S*),18(*R*)‐EpETE stereoselectively produced by BM‐3 suppresses CHS. We found that BM‐3 17(*S*),18(*R*)‐EpETE suppressed ear swelling (Figure 3A), and histologic analysis confirmed that BM‐3 17(*S*),18(*R*)‐EpETE decreased inflammation in the skin (Figure 3B). We previously reported that commercially available Cayman (±)17,18‐EpETE ameliorated CHS by inhibiting pseudopod formation in neutrophils and thus suppressed their infiltration into inflamed skin.^10^ Consistent with these previous findings, treatment with BM‐3 17(*S*),18(*R*)‐EpETE decreased the number of neutrophils in inflamed ears (Figure 4A). Histologic analysis confirmed the decreased number of neutrophils in the skin that had been treated with BM‐3 17(*S*),18(*R*)‐EpETE (Figure 4B). In addition, we examined dose‐dependent effects of BM‐3 17(*S*),18(*R*)‐EpETE in CHS by evaluating ear swelling and neutrophil numbers. Both ear swelling and neutrophil numbers were decreased at the dose of 1 µg and 100 ng/animal, but the anti‐inflammatory effects were hardly observed at the dose of 10 ng/animal (Figure S1A,B). Therefore, BM‐3 17(*S*),18(*R*)‐EpETE was shown to be effective at the dose more than 100 ng/animal.

### 3.4 BM‐3 17(*S*),18(*R*)‐EpETE inhibits neutrophil pseudopod formation in a GPR40‐dependent manner

**Figure 5:**
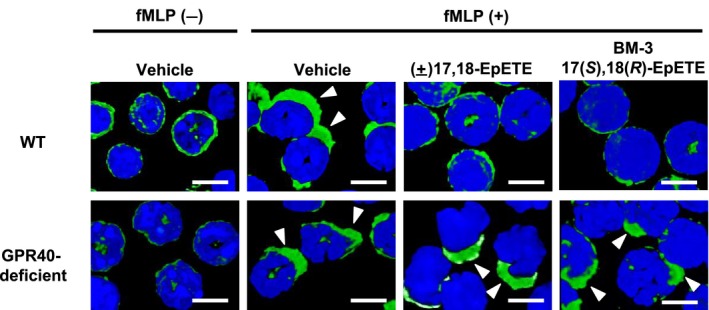
BM‐3 17(*S*),18(*R*)‐EpETE inhibits fMLP‐induced neutrophil pseudopod formation through GPR40. Neutrophils were isolated from the bone marrow of WT and GPR40‐deficient mice and stained with 4ʹ,6‐diamidino‐2‐phenylindole and Acti‐stain 488–phalloidin for analysis of pseudopod formation. Neutrophils were incubated with commercially available Cayman (±)17,18‐EpETE (100 nmol/L), BM‐3 17(*S*),18(*R*)‐EpETE (100 nmol/L), or vehicle only (0.03% ethanol solution) for 15 min before stimulation with fMLP (1 µmol/L) for 2 min. Data are representative of two to four independent experiments. Arrowheads indicate pseudopods. Bars, 5 μm

We next evaluated the inhibitory effect of BM‐3 17(*S*),18(*R*)‐EpETE on pseudopod formation in neutrophils isolated from bone barrow. We found that fMLP‐induced pseudopod formation was inhibited by treatment with BM‐3 17(*S*),18(*R*)‐EpETE (Figure 5). In agreement with our previous study showing that this effect was mediated through GPR40,^10^ the inhibitory effect of BM‐3 17(*S*),18(*R*)‐EpETE was absent when the neutrophils were prepared from GPR40‐deficient mice. We also found that BM‐3 17(*S*),18(*R*)‐EpETE inhibited LTB_4_‐induced pseudopod formation in a GPR40‐dependent manner (Figure S2). In addition, we found that the expression levels of chemokines such as *Cxcl1*, *Cxcl2*, *Cxcl9*, and *Cxcl10*, which are mainly produced by keratinocytes in the skin and are involved in the induction of CHS,^20^, ^25^, ^26^ were not changed by BM‐3 17(*S*),18(*R*)‐EpETE treatment (Figure S3). We further found that BM‐3 17(*S*),18(*R*)‐EpETE did not inhibit the expression of *Ifn‐γ* (Figure S3). These observations are consistent with our previous report that (±)17,18‐EpETE did not affect the number of IFN‐γ^+^ T cells in the ear of CHS.^10^ Taken together, these results demonstrated that BM‐3 17(*S*),18(*R*)‐EpETE acts on neutrophil selectively.

### 3.5 Dose‐dependent effects of BM‐3 17(*S*),18(*R*)‐EpETE, RvE1, and 18‐HEPE on the pseudopod formation

We compared the effectiveness of BM‐3 17(*S*),18(*R*)‐EpETE with EPA‐derived fatty acid metabolites, RvE1 and 18‐HEPE, for inhibition of pseudopod formation (Figures S4 and S5). When neutrophils were stimulated with fMLP, we found that all three types of lipids inhibited pseudopod formation at the dose of 1 μmol/L and 100 nmol/L. BM‐3 17(*S*),18(*R*)‐EpETE showed inhibitory effects at the dose of 10 nmol/L, while RvE1 and 18‐HEPE lost their activities. BM‐3 17(*S*),18(*R*)‐EpETE also lost its activity at the dose of 1 nmol/L.

In the case of LTB_4_‐induced pseudopod formation, we found that all three types of lipids inhibited pseudopod formation at the dose of 1 μmol/L and 100 nmol/L. In contrast to the case of fMLP, LTB_4_‐induced pseudopod formation was not inhibited by BM‐3 17(*S*),18(*R*)‐EpETE at the dose of 10 nmol/L, while RvE1 and 18‐HEPE showed inhibitory effects. Both RvE1 and 18‐HEPE lost its activity at the dose of 1 nmol/L. These results indicated that EPA‐derived bioactive lipid mediators of BM‐3 17(*S*),18(*R*)‐EpETE, RvE1, and 18‐HEPE all possess anti‐inflammatory activity by inhibiting neutrophil pseudopod formation.

## 4 DISCUSSION

Actin polymerization and neutrophil migration are induced by different kinds of chemoattractants. The fMLP promotes primary neutrophil migration in response to bacterial infection, while LTB_4_, which is secreted by neutrophils, promotes secondary neutrophil migration.^27^, ^28^ Previously, we found that (±)17,18‐EpETE reduced fMLP‐induced pseudopod formation by inhibiting Rac activation in a GPR40‐dependent manner.^10^ In the current study, we found that BM‐3 17(*S*),18(*R*)‐EpETE inhibited both fMLP‐ and LTB_4_‐induced pseudopod formation, and RvE1 and 18‐HEPE also inhibited both fMLP‐ and LTB_4_‐induced pseudopod formation. Consistent with these findings, a previous report showed that RvE1 and 18‐HEPE reduced neutrophil transmigration.^15^ Our current findings indicated that anti‐inflammatory mechanism of these lipid mediators might be different. As a possible mechanism, it was reported that these lipid mediators utilized different receptors. RvE1 acts as an agonist of ChemR23, and it also functions as an antagonist of the LTB_4_ receptor, BLT1.^29^ Activation of ChemR23‐mediated pathway reportedly suppresses fMLP‐induced neutrophil chemotaxis.^30^, ^31^ RvE1 also blocked LTB_4_‐induced pseudopod formation through BLT1 antagonistic activity in DC.^4^, ^29^ The specific receptor of 18‐HEPE was not identified, however, 18‐HEPE is a precursor of RvE1, which is mediated by 5‐lipoxygenase expressed by neutrophils.^15^ Therefore, it is plausible that 18‐HEPE inhibited neutrophil pseudopod formation by functioning as RvE1 precursor. The evidence suggested that 17(*S*),18(*R*)‐EpETE inhibited neutrophil pseudopod formation by GPR40‐mediated pathway, while 18‐HEPE and RvE1 used ChemR23 and/or BLT1 as functional receptors.

The use of single‐enantiomer medicines can potentially lead to simpler and more selective pharmacologic profiles, because the enantiomers of a chiral compound may differ significantly in their bioavailability.^32^ For example, one enantiomer may be responsible for the therapeutic effects of a medicine, whereas the other enantiomer may be inactive or contribute to undesirable side effects.^32^ For example, the racemate of (*S*)‐ and (*R*)‐thalidomide was introduced as a sedative medicine in the late 1950s, but it was withdrawn due to teratogenicity of (*S*)‐thalidomide, indicating the importance of stereoselective production of candidate medicines.^33^, ^34^ In another case, the (*S*)‐ and (*R*)‐enantiomers of salbutamol exert different effects: the (*R*)‐enantiomer of salbutamol binds the B_2_‐adrenergic receptor with greater affinity than the (*S*)‐enantiomer and is responsible for salbutamol's bronchodilation activity.^35^, ^36^ Therefore, treatment by using a single enantiomer is recommended to avoid the undesired effects of the other isomer and to optimize the therapeutic effects of the medicine. The industrial production of chemical compounds has several known disadvantages, including low catalytic efficiency; lack of stereoselectivity; the need for extreme conditions such as high temperature, low pH, and high pressure; and the generation of organic solvent waste.^37^ Conversely, enzymatic reactions work under mild conditions, have low environmental impact, and yield high stereoselectivity.^38^ Therefore, many industrial processes use enzymes or whole microorganisms for the synthesis of chemical compounds.^37^

In this study, we found that BM‐3 17(*S*),18(*R*)‐EpETE stereoselectively showed anti‐inflammatory activity in CHS. Conversely, it is reported that 17(*R*),18(*S*)‐EpETE—but not 17(*S*),18(*R*)‐EpETE—is a potent vasodilator and stimulates calcium‐activated potassium channels, which lead to the relaxation of rat cerebral artery vascular smooth muscle cells.^12^, ^13^ The stereoselective‐dependent differences in these isomers’ anti‐inflammatory and relaxing effects on arteries are likely to be due to the use of different receptors. Hence, the 17(*S*),18(*R*)‐EpETE–GPR40 axis suppresses CHS, whereas the 17(*R*),18(*S*)‐EpETE–calcium‐activated potassium‐channel axis achieves arterial relaxation.^10^, ^12^, ^13^ Therefore, using 17(*S*),18(*R*)‐EpETE as a single‐enantiomer therapy might decrease the risk of side effects due to vasodilatory activity, such as a rapid decrease in blood pressure and increased skin redness.

We previously found that dietary linseed oil, which contains large amounts of α‐linolenic acid, a precursor of EPA and DHA, increases the amount of ω3 PUFA‐derived metabolites in the body.^9^ In particular, we found that 17,18‐EpETE was a major product in the gut, although which isomer(s) is generated is unknown.^9^ Therefore, the activity of CYPs in the gut is likely to be higher than that of cyclooxygenase and lipoxygenase. Furthermore, CYPs also are present in the liver, lung, kidney, brain, and skin, suggesting that dietary ω3 PUFAs lead to the production of 17,18‐EpETE in various sites in the body.^39^, ^40^ However, despite the many types of CYPs that exist, it is reported that only five types of CYPs can convert EPA into 17,18‐EpETE in mice, and only nine types have this function in humans.^24^, ^41^, ^42^ Depending on the CYP type, 17,18‐EpETE is produced in a stereoselective or non‐stereoselective manner. For example, Cyp1a2 in mice and CYP2E1 in humans selectively generate 17(*R*),18(*S*)‐EpETE, whereas Cyp4f18 in mice and CYP2D6 in humans—like BM‐3—selectively generate 17(*S*),18(*R*)‐EpETE.^24^, ^41^ In contrast, other CYPs, such as Cyp4a12a, Cyp4a12b, and Cyp2c50 in mice and CYP1A1, CYP2C19, CYP2C9, CYP2J2, CYP3A4, CYP2C18, and CYP2C8 in humans, produce both 17(*R*),18(*S*)‐EpETE and 17(*S*),18(*R*)‐EpETE.^24^, ^41^

CYPs harbor gene polymorphisms, which cause different enzymatic activities among individuals.^40^, ^43^, ^44^ Therefore, differences in the production levels and profiles of lipid metabolites including 17(*S*),18(*R*)‐EpETE might reflect differences in CYP activity or expression level. For example, the expression level of Cyp1a2 is upregulated by the ligand‐activated transcription factor aromatic hydrocarbon receptor (AhR).^45^ AhR can be activated by naturally occurring indoles, such as indole‐3‐carbinol, which are abundant in various vegetables, including broccoli, cabbage, and cauliflower, suggesting that production levels of 17(*R*),18(*S*)‐EpETE might be modulated through diet.^46^, ^47^ In another example, the expression levels of the Cyp4a subfamily including Cyp4a10, Cyp4a12, and Cyp4a14 are upregulated by PPARα activation.^48^ Because PUFAs, polyphenols, and carotenoids are all potent PPARα ligands, expression levels of Cyp4a subfamily members might be regulated by dietary components which in turn control the production of 17(*S*),18(*R*)‐EpETE and 17(*R*),18(*S*)‐EpETE.^49^, ^50^, ^51^, ^52^, ^53^, ^54^ These findings indicate that the efficacy of dietary ω3 PUFA can be regulated by various food components through the induction of CYP expression, followed by production of 17,18‐EpETE. Therefore, the direct intake of bioactive lipid metabolites—rather than of the precursor molecules α‐linolenic acid, EPA, and DHA—may be a better way to obtain desired biologic effects, such as anti‐inflammatory and anti‐allergic activities, by avoiding the potential genetic and food‐associated effects on the efficacy of dietary ω3 PUFAs in the regulation of health and diseases.^1^, ^2^, ^9^, ^55^

Because microorganisms metabolize fatty acids, microorganisms can affect the lipid profile. In particular, microorganisms are used for the production of fermented foods, which may often contain abundant amounts of lipid metabolites. For example, *Bacillus* bacteria, including *B. megaterium*, are used for the production of fermented foods, such as the soybean products natto and miso. Therefore, the production level of lipid metabolites likely is affected not only by the enzymes in the body but also by enzymes derived from microorganisms in fermented food, suggesting that the production levels of 17(*S*),18(*R*)‐EpETE could be increased by eating fermented foods containing *Bacillus* bacteria.^56^ The incubation of EPA and *B. megaterium* homogenate reportedly yields 17,18‐EpETE and 18‐HEPE, whereas BM‐3 primarily catalyzes epoxidation and produces 17,18‐EpETE from EPA, thus suggesting that not only BM‐3, various types of CYPs in *B. megaterium* contribute to the production of 17,18‐EpETE and 18‐HEPE.^15^, ^16^ Therefore, screening CYP‐containing microorganisms that metabolize ω3 PUFAs for SPMs is required to obtain the desired metabolites. For the production of bioactive lipid metabolites, lipid mediators, and bacteria for consumption should be selected depending on the disease involved, to maximize health and disease treatment.

In conclusion, the present study showed enzymatically produced 17(*S*),18(*R*)‐EpETE can reduce CHS. The 17(*S*),18(*R*)‐EpETE–GPR40 axis played a key role in the amelioration of CHS by inhibiting neutrophil migration. These results suggest that bacterial fermentation with BM‐3 activity is a promising tool for the stereoselective mass‐production of 17(*S*),18(*R*)‐EpETE.

## CONFLICT OF INTEREST

There are no conflict of interest to declare. 

## AUTHOR CONTRIBUTIONS

AS, TN, and JK designed the research and wrote the paper. AS, TN, SK, SP, NM, MS, SM, EN, and JO performed experiments, analyzed data, and discussed the results. TH, PT, SH, KH, and KK provided technical help and discussed the results.
