# Supplementary material for: 17(S),18(R)‐epoxyeicosatetraenoic acid generated by cytochrome P450 BM‐3 from Bacillus megaterium inhibits the development of contact hypersensitivity via G‐protein‐coupled receptor 40‐mediated neutrophil suppression
Source: FASEB Bioadv. 2019 Dec 24;2(1):59–71. doi: 10.1096/fba.2019-00061 (PMC6996328; doi:10.1096/fba.2019-00061)
Supplement: Supplementary file 1 [file FBA2-2-59-s001.pptx]

## Slide 1
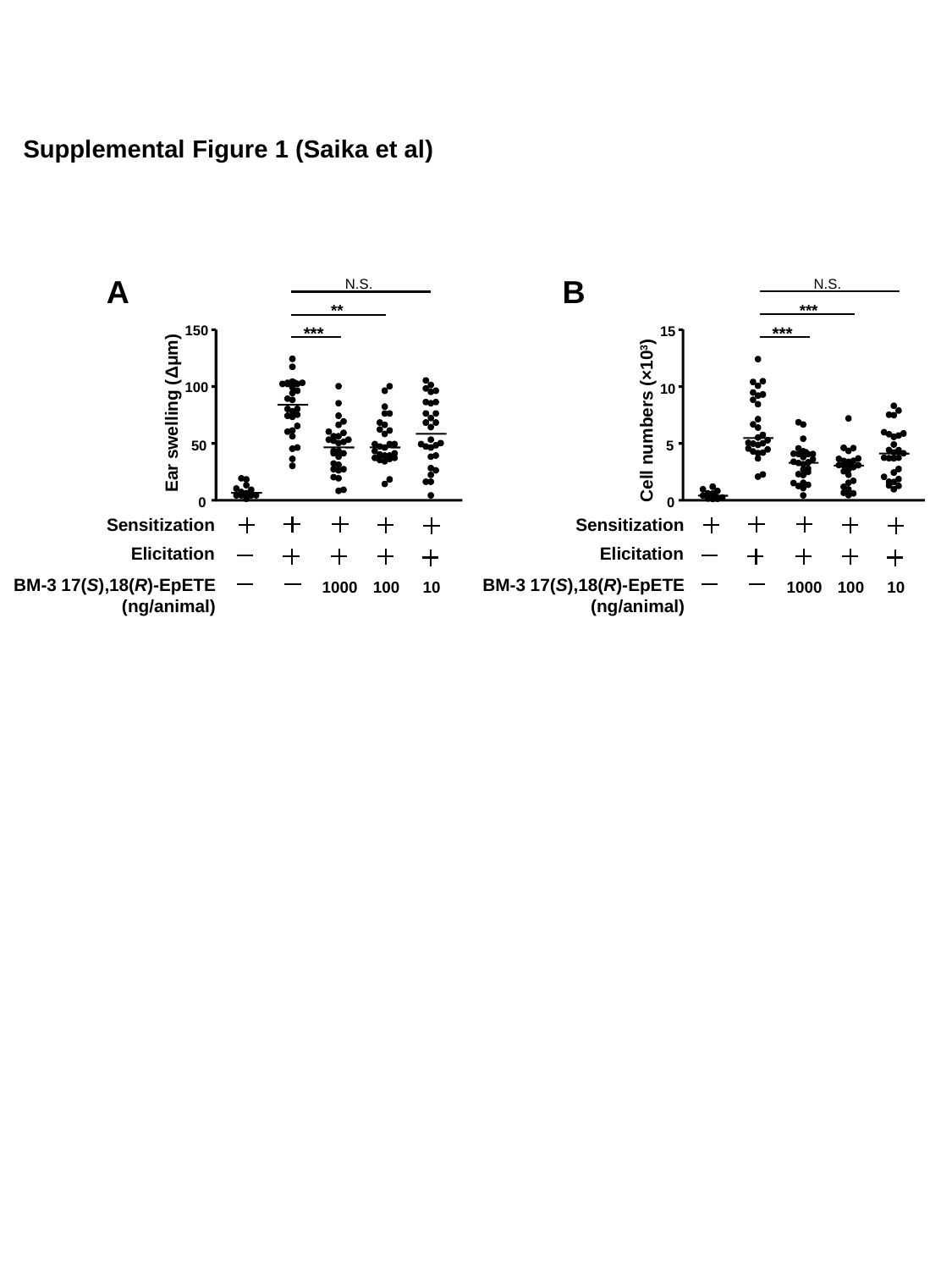

Supplemental Figure 1 (Saika et al)
A
B
N.S.
N.S.
***
**
***
***
150
100
Ear swelling (Δμm)
50
0
15
10
Cell numbers (×103)
5
0
Sensitization
Sensitization
Elicitation
Elicitation
BM-3 17(S),18(R)-EpETE
(ng/animal)
BM-3 17(S),18(R)-EpETE
(ng/animal)
1000
1000
100
10
100
10
