# Supplementary figures and images for: 17(S),18(R)‐epoxyeicosatetraenoic acid generated by cytochrome P450 BM‐3 from Bacillus megaterium inhibits the development of contact hypersensitivity via G‐protein‐coupled receptor 40‐mediated neutrophil suppression
Source: FASEB Bioadv. 2019 Dec 24;2(1):59–71. doi: 10.1096/fba.2019-00061 (PMC6996328; doi:10.1096/fba.2019-00061)

## Slide 1
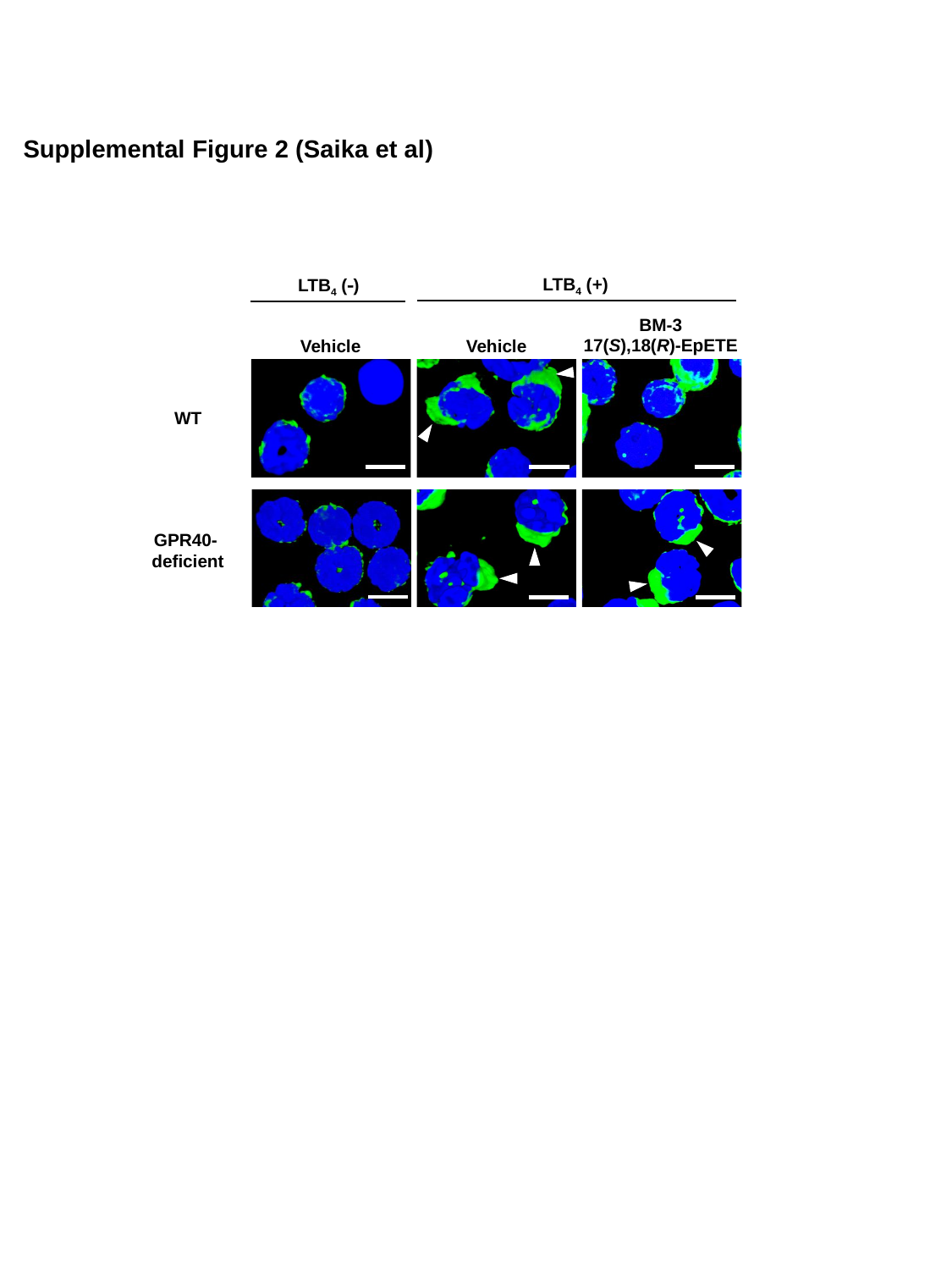

Supplemental Figure 2 (Saika et al)
LTB4 (+)
LTB4 (−)
BM-3
17(S),18(R)-EpETE
Vehicle
Vehicle
WT
GPR40-
deficient

Supplement: Supplementary file 2 [file FBA2-2-59-s002.pptx]
