# Supplementary material for: 17(S),18(R)‐epoxyeicosatetraenoic acid generated by cytochrome P450 BM‐3 from Bacillus megaterium inhibits the development of contact hypersensitivity via G‐protein‐coupled receptor 40‐mediated neutrophil suppression
Source: FASEB Bioadv. 2019 Dec 24;2(1):59–71. doi: 10.1096/fba.2019-00061 (PMC6996328; doi:10.1096/fba.2019-00061)
Supplement: Supplementary file 3 [file FBA2-2-59-s003.pptx]

## Slide 1
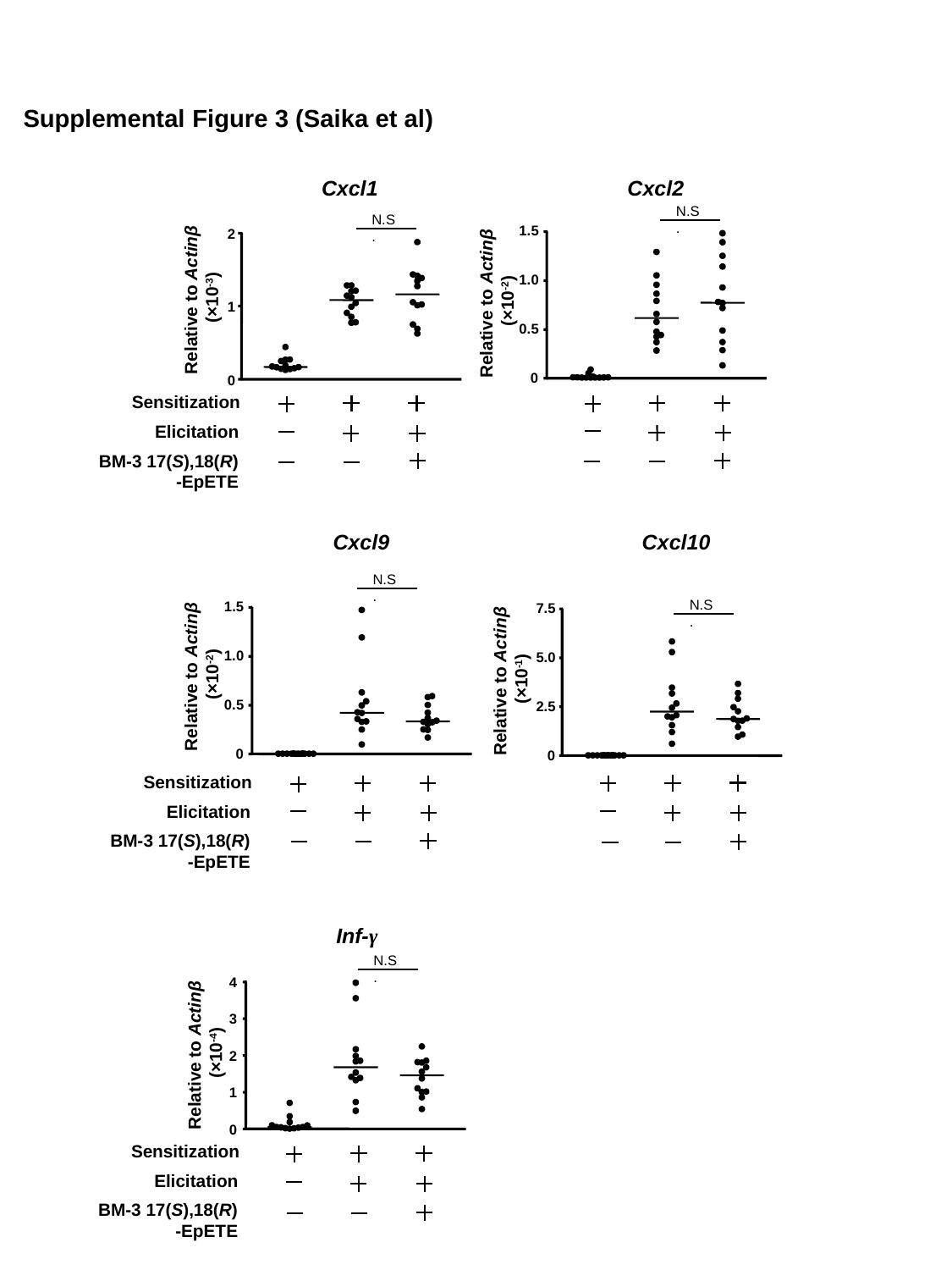

Supplemental Figure 3 (Saika et al)
Cxcl1
2
Relative to Actinβ
 (×10-3)
1
0
Cxcl2
1.5
1.0
Relative to Actinβ
 (×10-2)
0.5
0
N.S.
N.S.
Sensitization
Elicitation
BM-3 17(S),18(R)
-EpETE
Cxcl9
1.5
1.0
Relative to Actinβ
(×10-2)
0.5
0
Cxcl10
7.5
5.0
Relative to Actinβ
 (×10-1)
2.5
0
N.S.
N.S.
Sensitization
Elicitation
BM-3 17(S),18(R)
-EpETE
Inf-γ
N.S.
4
3
Relative to Actinβ
(×10-4)
2
1
0
Sensitization
Elicitation
BM-3 17(S),18(R)
-EpETE
