# Supplementary material for: 17(S),18(R)‐epoxyeicosatetraenoic acid generated by cytochrome P450 BM‐3 from Bacillus megaterium inhibits the development of contact hypersensitivity via G‐protein‐coupled receptor 40‐mediated neutrophil suppression
Source: FASEB Bioadv. 2019 Dec 24;2(1):59–71. doi: 10.1096/fba.2019-00061 (PMC6996328; doi:10.1096/fba.2019-00061)
Supplement: Supplementary file 5 [file FBA2-2-59-s005.pptx]

## Slide 1
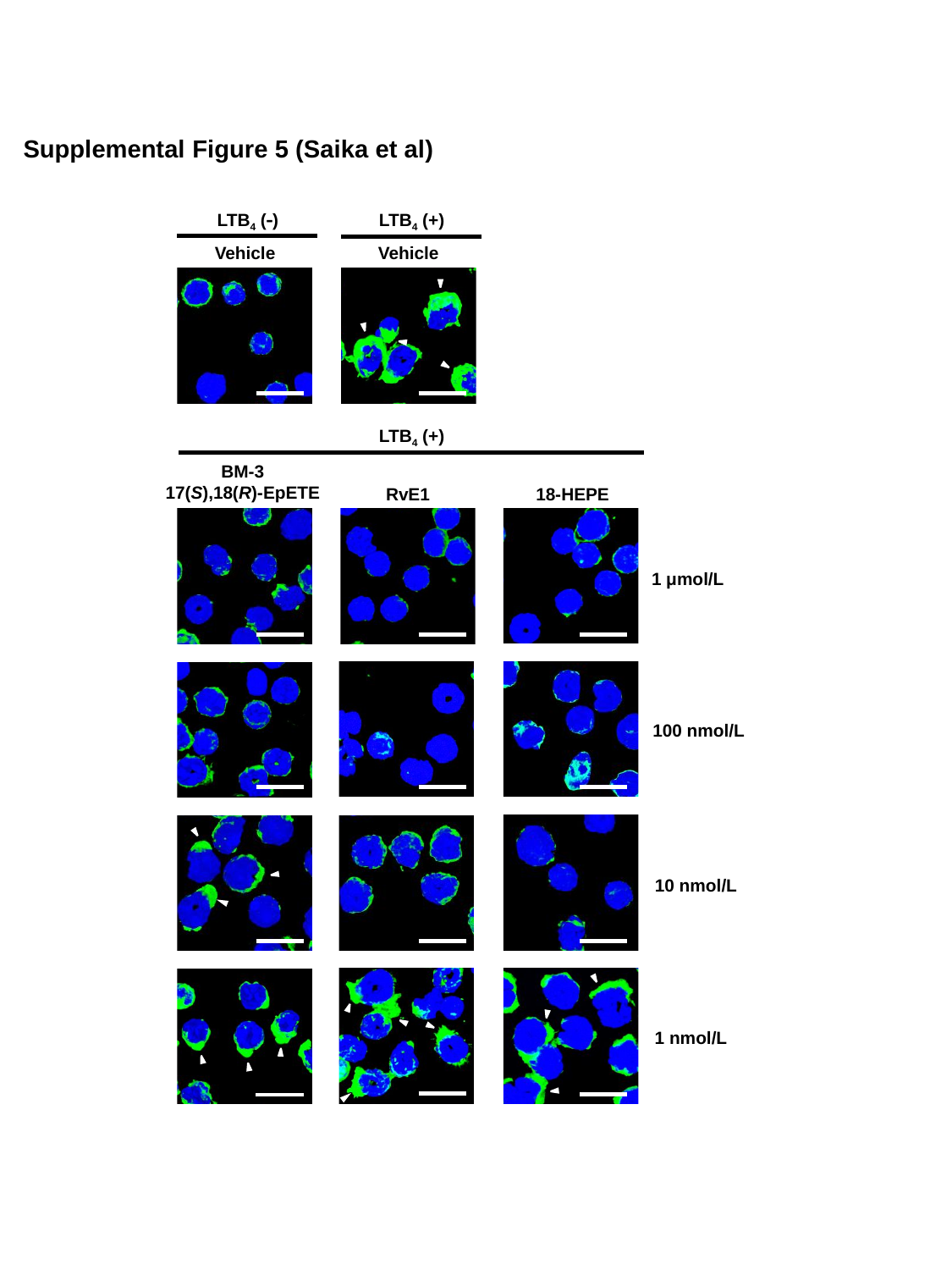

Supplemental Figure 5 (Saika et al)
LTB4 (−)
LTB4 (+)
Vehicle
Vehicle
LTB4 (+)
BM-3
17(S),18(R)-EpETE
RvE1
18-HEPE
1 μmol/L
100 nmol/L
10 nmol/L
1 nmol/L
