# Supplementary material for: 17(S),18(R)‐epoxyeicosatetraenoic acid generated by cytochrome P450 BM‐3 from Bacillus megaterium inhibits the development of contact hypersensitivity via G‐protein‐coupled receptor 40‐mediated neutrophil suppression
Source: FASEB Bioadv. 2019 Dec 24;2(1):59–71. doi: 10.1096/fba.2019-00061 (PMC6996328; doi:10.1096/fba.2019-00061)
Supplement: Supplementary file 6 [file FBA2-2-59-s006.pdf]

Supplemental figure legends

Supplemental Figure 1

Dose dependent effects of BM-3 17(*S*),18(*R*)-EpETE in CHS

Mice were injected intraperitoneally with either 1 μg/animal, 100 ng/animal or 10 ng/animal of BM-3 17(*S*),18(*R*)-EpETE, or vehicle only on days 0 and 5 at 30 minutes before DNFB application. (A) Ear swelling was evaluated on day 7. (B) On day 7, flow cytometry was used to count the cell number of $Ly6G^+$ $CD11b^+$ neutrophils. Data are combined from 4 independent experiments, and each point represents data from an individual mouse. Horizontal bars among data points indicate median values. The statistical significance was evaluated by using one-way ANOVA; **, $P < 0.01$, ***, $P < 0.001$, N.S., not significant.

Supplemental Figure 2

BM-3 17(*S*),18(*R*)-EpETE inhibits LTB_4-induced neutrophil pseudopod formation through GPR40

Neutrophils were isolated from the bone marrow of WT and GPR40-deficient mice and stained with 4′,6-diamidino-2-phenylindole and Acti-stain 488–phalloidin for analysis of pseudopod formation. Neutrophils were incubated with BM-3 17(*S*),18(*R*)-EpETE (100 nmol/L), or vehicle only (0.03% ethanol solution) for 15 minutes before stimulation with LTB_4 (100 nmol/L) for 2 minutes. Data are representative of 2 independent experiments. Arrowheads indicate pseudopods. Bars, 5 μm.

Supplemental Figure 3

BM-3 17(*S*),18(*R*)-EpETE does not affect chemokines and cytokine gene expression

Mice were injected intraperitoneally with 100 ng/animal of BM-3 17(*S*),18(*R*)-EpETE, or vehicle only on days 0 and 5 at 30 minutes before DNFB application. On day 7, ear tissues were homogenized for isolation of mRNA, and quantitative RT-PCR analysis was performed to measure *Cxcl1*, *Cxcl2*, *Cxcl9*, *Cxcl10*, *Ifn-γ*, and *Actinβ* expression, which was normalized to the expression of *Actinβ*. Data are combined from 2 independent experiments, and each point represents data from an individual mouse. Horizontal bars among data points indicate median values. The statistical significance was evaluated by using one-way ANOVA; N.S., not significant.

Supplemental Figure 4

Dose dependent effects of BM-3 17(*S*),18(*R*)-EpETE, RvE1, and 18-HEPE on fMLP-induced neutrophil pseudopod formation

Neutrophils were isolated from the bone marrow of WT mice and stained with 4',6-diamidino-2-phenylindole and Acti-stain 488–phalloidin for analysis of pseudopod formation. Neutrophils were

incubated with BM-3 17(*S*),18(*R*)-EpETE, RvE1, or 18-HEPE (1 μmol/L, 100 nmol/L, 10 nmol/L, 1 nmol/L), or vehicle only (0.03% ethanol solution) for 15 minutes before stimulation with fMLP (1 μmol/L) for 2 minutes. Data are representative of 2 independent experiments. Arrowheads indicate pseudopods. Bars, 10 μm.

Supplemental Figure 5

Dose dependent effects of BM-3 17(*S*),18(*R*)-EpETE, RvE1, and 18-HEPE on LTB_4-induced neutrophil pseudopod formation

Neutrophils were isolated from the bone marrow of WT mice and stained with 4',6-diamidino-2-phenylindole and Acti-stain 488–phalloidin for analysis of pseudopod formation. Neutrophils were incubated with BM-3 17(*S*),18(*R*)-EpETE, RvE1, or 18-HEPE (1 μmol/L, 100 nmol/L, 10 nmol/L, 1 nmol/L), or vehicle only (0.03% ethanol solution) for 15 minutes before stimulation with LTB_4 (100 nmol/L) for 2 minutes. Data are representative of 2 independent experiments. Arrowheads indicate pseudopods. Bars, 10 μm.
